# Effect of *Lactobacillus plantarum* TWK10 on Exercise Physiological Adaptation, Performance, and Body Composition in Healthy Humans

**DOI:** 10.3390/nu11112836

**Published:** 2019-11-19

**Authors:** Wen-Ching Huang, Mon-Chien Lee, Chia-Chia Lee, Ker-Sin Ng, Yi-Ju Hsu, Tsung-Yu Tsai, San-Land Young, Jin-Seng Lin, Chi-Chang Huang

**Affiliations:** 1 Department of Exercise and Health Science, National Taipei University of Nursing and Health Sciences, Taipei 11219, Taiwan; wenching@ntunhs.edu.tw; 2 Graduate Institute of Sports Science, National Taiwan Sport University, Taoyuan 33301, Taiwan; 1061304@ntsu.edu.tw (M.-C.L.); ruby780202@ntsu.edu.tw (Y.-J.H.); 3 Culture Collection & Research Institute, SYNBIO TECH INC., Kaohsiung 82151, Taiwan; cclee@synbiotech.com.tw (C.-C.L.); ks-ng@synbiotech.com.tw (K.-S.N.); s333@synbiotech.com.tw (S.-L.Y.); 4 Department of Food Science, Fu Jen Catholic University, New Taipei City 24205, Taiwan; tytsai@mail.fju.edu.tw

**Keywords:** *Lactobacillus plantarum*, probiotics, endurance performance, body composition, muscle mass

## Abstract

Probiotics have been rapidly developed for health promotion, but clinical validation of the effects on exercise physiology has been limited. In a previous study, *Lactobacillus plantarum* TWK10 (TWK10), isolated from Taiwanese pickled cabbage as a probiotic, was demonstrated to improve exercise performance in an animal model. Thus, in the current study, we attempted to further validate the physiological function and benefits through clinical trials for the purpose of translational research. The study was designed as a double-blind placebo-controlled experiment. A total of 54 healthy participants (27 men and 27 women) aged 20–30 years without professional athletic training were enrolled and randomly allocated to the placebo, low (3 × 10^10^ colony forming units (CFU)), and high dose (9 × 10^10^ CFU) TWK10 administration groups (*n* = 18 per group, with equal sexes). The functional and physiological assessments were conducted by exhaustive treadmill exercise measurements (85% VO_2max_), and related biochemical indices were measured before and after six weeks of administration. Fatigue-associated indices, including lactic acid, blood ammonia, blood glucose, and creatinine kinase, were continuously monitored during 30 min of exercise and a 90 min rest period using fixed intensity exercise challenges (60% VO_2max_) to understand the physiological adaptation. The systemic inflammation and body compositions were also acquired and analyzed during the experimental process. The results showed that TWK10 significantly elevated the exercise performance in a dose-dependent manner and improved the fatigue-associated features correlated with better physiological adaptation. The change in body composition shifted in the healthy direction for TWK10 administration groups, especially for the high TWK10 dose group, which showed that body fat significantly decreased and muscle mass significantly increased. Taken together, our results suggest that TWK10 has the potential to be an ergogenic aid to improve aerobic endurance performance via physiological adaptation effects.

## 1. Introduction

Fatigue syndromes can result from pathological and nonpathological impacts [1]. Pathological fatigue, such as chronic fatigue syndrome (CFS), can be caused by cancer, infection, endocrinological imbalance, immune problems, and psychosomatic diseases, and CFS may not be an independent and unique disease [2]. Nonpathological/physiological fatigue can be further characterized as induced via central and peripheral mechanisms [3] and both mechanisms play important roles in the physiological effects during exercise processes, types, intensities, and duration [4]. Exercise-induced muscle damage (EIMD) generally occurs due to overload if unaccustomed to exercise, causing inflammation, pain, soreness, and muscular fitness decrease [5]. Nutritional supplementation has the potential to mitigate EIMD [6]. Thus, strategies including nutrition intervention as well as training programs [7] and physical therapies [8] have been developed to improve physiological fatigue, adaptation, and performance. Optimized physiological adaption could be beneficial for health promotion from the perspective of exercise and nutrition.

Exercise promotes energy, harvesting [9] the gut microbiota ferments complex dietary polysaccharides into short chain fatty acids (SCFAs), which can be used as an energy source for liver and muscle cells and improves endurance performance by maintaining blood glucose for a long period of time. The resulting SCFAs appear to regulate neutrophil function and migration, reduce colonic mucosal permeability, inhibit inflammatory cytokines, and control the redox environment in cells, which may help enhance muscle renewal and adaptability, improve exercise performance, and delay fatigue symptoms [10]. Many studies have indicated that, in addition to exercise, supplementation of prebiotics or probiotics can increase intestinal SCFA levels [11], including acetic acid, n-butyric acid, and propionic acid. These can regulate the energy balance of the host and increase the use of nutrients [12]. Regular use of probiotics can positively alter the amount and structure of the gut flora and may affect the immune function of individuals after exercise as well as the proliferation, function, and protection of intestinal epithelial cells [10]. Probiotics are food supplements that contain living microorganisms, especially lactic acid bacteria, which, if provided in sufficient amounts, can benefit the health of the host [13].

The effects of probiotics on exercise performance may vary depending on the probiotics used. Different strains have different mechanisms of action, which have not been confirmed. Previous studies have demonstrated the effects of probiotics on exercise performance and associated physiological responses. A mixture of two *Lactobacillus* strains exerted strong antioxidant activity that neutralized athlete oxidative stress induced by intense exercise training after four weeks of ingestion [14]. *Bacillus subtilis* DE111 supplementation attenuated circulating TNF-α in athletes but had no effect on body composition, performance, hormonal status, or gut permeability [15]. Multi-strain probiotic supplementation had limited to large effects on gastrointestinal structural integrity, endotoxin translocation, and immune modulation, and may have an additive effect in enhancing exercise performance in hot environments [16]. An adequate probiotic supplementation could improve intestinal barrier function, redox hemostasis, and low-grade inflammation in trained men under sustained exercise stress [17]. Most studies related to probiotics and athletic performance are primarily aimed at athletes; studies on healthy untrained humans are lacking.

In the current study, we administered *Lactobacillus plantarum* TWK10 probiotics (TWK10) to healthy subjects without an athletic background for six weeks. We examined the effects of TWK10 on fatigue-associated indices, exercise performance, and body composition. Based on our previous animal and elite athletic studies [18,19], TWK10 produces different physiological effects on health promotion; safety was also evaluated via current clinical experimental designs.

## 2. Materials and Methods

### 2.1. Probiotics

TWK10, a plant-derived the *Lactobacillus* strain, was isolated from Taiwanese pickled cabbage [18]. The TWK10 in the current study was cultivated and produced by SYNBIO TECH INC. (Kaohsiung, Taiwan) in capsule format with indicated doses. Each TWK10 (lyophilized bacterial powder) indicated capsule contained either 1 × 10^10^ or 3 × 10^10^ colony-forming units (CFU) of TWK10 and was standardized with maltodextrin and microcrystalline cellulose. The ingredients of the placebo capsule were similar to the TWK10 capsule but without the addition of TWK10. The designed dosage was three capsules per day after meals.

### 2.2. Volunteers

The test volunteers were 54 healthy participants (27 men and 27 women; age, 20–30 years old) without professional athletic training. Volunteers were excluded from this study if they had any known disorders, including heart/cardiopulmonary disease, diabetes, thyroid disease, hypogonadism, hepatorenal disease, musculoskeletal disorder, neuromuscular/neurological disease, autoimmune disease, cancer, peptic ulcers, ulcerative colitis, or Crohn’s disease. All volunteers were prohibited from taking probiotics, prebiotics, fermented products (yogurt or foods), vitamins, minerals, herbal extracts, dietary supplements for exercise and athletic performance, and antibiotics to avoid unnecessary interference during the experimental period. All volunteers provided written informed consent before participation. The study was reviewed and approved by the Institutional Review Board of Landseed International Hospital (Taoyuan, Taiwan; LSHIRB No. 18-004-A2). Volunteers were advised to maintain their normal lifestyle and physical activities. The volunteers were also asked to maintain their usual diets, and their recorded caloric consumptions in the before- and after-administration periods were assessed by dietitians. The basic demographic profiles and characteristics of the volunteers are shown in Table 1.

### 2.3. Experimental Design

We adopted a double-blind test in which the volunteers, based on their basal maximal oxygen consumption (VO_2max_), were equally assigned to one of three groups: A placebo group and 1× TWK10 (low dose; 3 × 10^10^ CFU/day) and 3× TWK10 (high dose; 9 × 10^10^ CFU/day) groups (18 subjects in each group with sexes equal). Maximal oxygen consumption and exercise performance were evaluated with a treadmill (Pulsar, h/p/cosmos, Nussdorf-Traunstein, Germany) and an auto respiratory analyzer Vmax 29c (Sensor Medics, Yorba Linda, CA, USA). Heart rate (HR) was also monitored using a Polar heart rate device. The speed of the treadmill ranged from 7.2 km/h and increased 1.8 km/h every 2 min until volitional fatigue in accordance with the Bruce protocol [20]. Oxygen consumption was considered maximum when the respiratory exchange ratio (the volume ratio of carbon dioxide produced to oxygen consumed, VCO_2_/VO_2_) was higher than 1.10 and the maximum heart rate was achieved (maximum heart rate = 210 − age). The three highest VO_2max_ values were averaged to obtain the VO_2max_ of the individual volunteer.

Basic characteristics, including body height, body weight, body mass index (BMI), VO_2max_, and caloric intake, were recorded before and after administration as references. Volunteers were notified through the LINE group communication app (LINE Taiwan, Taipei City, Taiwan) before every meal to administer either the placebo or TWK10 for 6 weeks. Volunteers were also told to avoid any strenuous physical activities for 3 days before the initial exercise test for VO_2max_ assessment.

**Figure 1:**
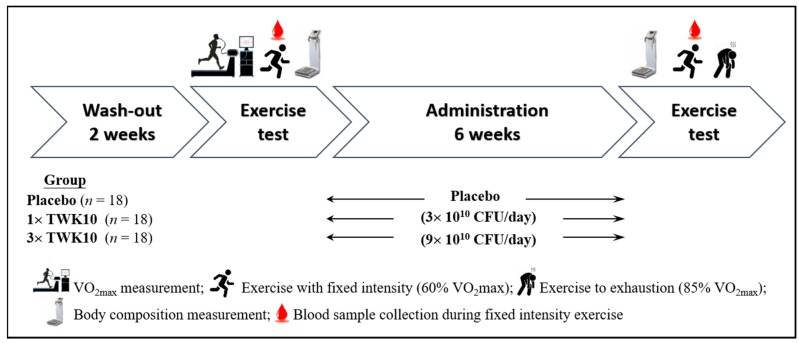
Experimental design. We adopted a double-blind test in which the subjects (18 subjects; men = 9, women = 9 in each group) were assigned to three groups: Placebo, 1× TWK10 (low dose; 3 × 10^10^ CFU/day) and 3× TWK10 (high dose; 9 × 10^10^ CFU/day). Subjects were administered with the indicated dosage of *Lactobacillus plantarum* TWK10 or placebo for six weeks. The individual VO_2max_ was measured prior to experiments for exercise intensity calibration, and the physiological adaptation effects were determined before and after administration using biochemical assessments.

The individual basal VO_2max_ during pretest was used as a reference to adjust the individually appropriate exercise intensity to measure physiological adaptation (60% VO_2max_) and exhaustive endurance (85% VO_2max_). Measurement was assessed using a warm-up stage for 5 min, followed by a test on the treadmill at 60% or 85% VO_2max_ workload. The exercise capacity and individual intensity adjustment followed previously reported standard protocols for evaluation of intervention effects [21]. To predict signs of exhaustion, the oxygen consumption, HR, and Borg’s rating of perceived exertion (RPE) were monitored every 5 min during the submaximal endurance exercise, and the sustained exercise duration was recorded as the endurance index. The detailed formula for intensity adjustment was modified from a previous study [19]. The experimental procedure is presented in Figure 1.

### 2.4. Clinical Biochemistry and Hematology Analysis

For assessment of fatigue-related indices, volunteers fasted for at least 8 h before the 60% VO_2max_ fixed intensity exercise challenge. Blood samples were collected with an arm venous catheter at indicated time points during exercise and recovery periods, including baseline (0), 5 (E5), 10 (E10), 15 (E15), and 30 (E30) min during the exercise phase, and 20 (R20), 40 (R40), 60 (R60), and 90 (R90) min in the recovery phase. Serum lactate, ammonia, glucose, and creatinine kinase (CK) were assessed for monitoring physiological adaptation. Clinical biochemistry indices, aspartate transaminase (AST), alanine aminotransferase (ALT), blood urea nitrogen (BUN), creatinine, and uric acid (UA) were also measured for physiological status before and after 6 weeks of administration. All biochemical indices were assessed using an autoanalyzer (Hitachi 7060, Tokyo, Japan). The complete blood count (CBC) profiles (MindrayBC-2800Vet, Shenzhen, China) were also analyzed at the 90 min time point in the recovery phase.

### 2.5. Body Composition

Body composition was measured by applying the multi-frequency principle with a bioelectrical impedance analyzer (BIA) on the InBody 770 (In-body, Seoul, Korea). This device provides 1, 5, 50, 260, 500, and 1000 kHz frequency screening in 60 s. For the measurement, the volunteers stood on the foothold electrodes after the subjects’ palms and soles were cleared; both hands held the sensing handle, and the volunteers kept their arms open and away from their body at a 30° angle without speaking and movement during the measurement period. The volunteers also fasted at least 8 h prior to the tests, which were performed twice: Before and after administration, with the same measuring interval before the physiological adaptation (60% VO_2max_) exercise challenge.

### 2.6. Statistics

All data are expressed as the mean ± SD. Statistical analyses were performed using Prism 7.04 (GraphPad, San Diego, CA, USA). Within-group differences (before vs. after TWK10 administration) and multiple group comparison were analyzed using a paired Student’s *t*-test and the Tukey–Kramer test, respectively. The Kruskal–Wallis test was used for multiple non-parametric comparison, including the ratio of the body composition change, ratio of neutrophil count to lymphocyte count (NLR), and ratio of platelet to lymphocyte (PLR). Differences were considered statistically significant at *P* < 0.05.

## 3. Results

### 3.1. Effects of TWK10 on Endurance Performance

VO_2max_ was measured before the tests were conducted to evaluate baseline exercise capacity individually, and volunteers were equally assigned to placebo, 1× TWK10, and 3× TWK10 groups (Table 1). The baseline value of exhaustion time was calculated based on the individual VO_2max_ value and the following equation, which is based on ACSM’s Health-Related Physical Fitness Assessment Manual:

VO_2max_ (mL/kg/min) = 14.76 − (1.379 × T) + (0.451 × T2) − (0.012 × T3).

**Figure 2:**
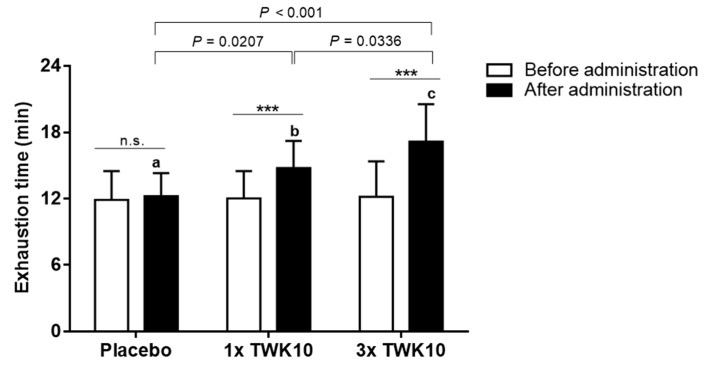
TWK10 improved exercise endurance performance in a dose-dependent manner. Endurance performance was examined under 85% VO_2max_ exercise intensity before and after TWK10 administration. Data are shown as the mean ± SD. Statistical differences among groups were analyzed using the Tukey–Kramer test, and different letters (a, b, c) indicate a significant difference at *P* < 0.05. Before and after administration effects were statistically analyzed using a paired Student’s *t*-test, *** *P* < 0.001.

From the within-group comparisons (Figure 2), we found that the exhaustion time of both TWK10 groups significantly increased after six weeks of administration, which was not observed in corresponding controls (paired *t*-test, placebo, *P* = 0.6247; 1× TWK10, *P* < 0.001; 3× TWK10, *P* < 0.001). The exhaustion time in the 1× TWK10 (*P* = 0.0207) and 3× TWK10 (*P* < 0.001) groups as compared to the placebo group after administration significantly increased, and the improvements were significant in the 3× TWK10 group compared to the 1× TWK10 group (*P* = 0.0336), indicating TWK10 significantly improved the endurance performance in a dose-dependent manner.

### 3.2. Effects of TWK10 on Physiological Adaptation and Biochemical Indices

**Figure 3:**
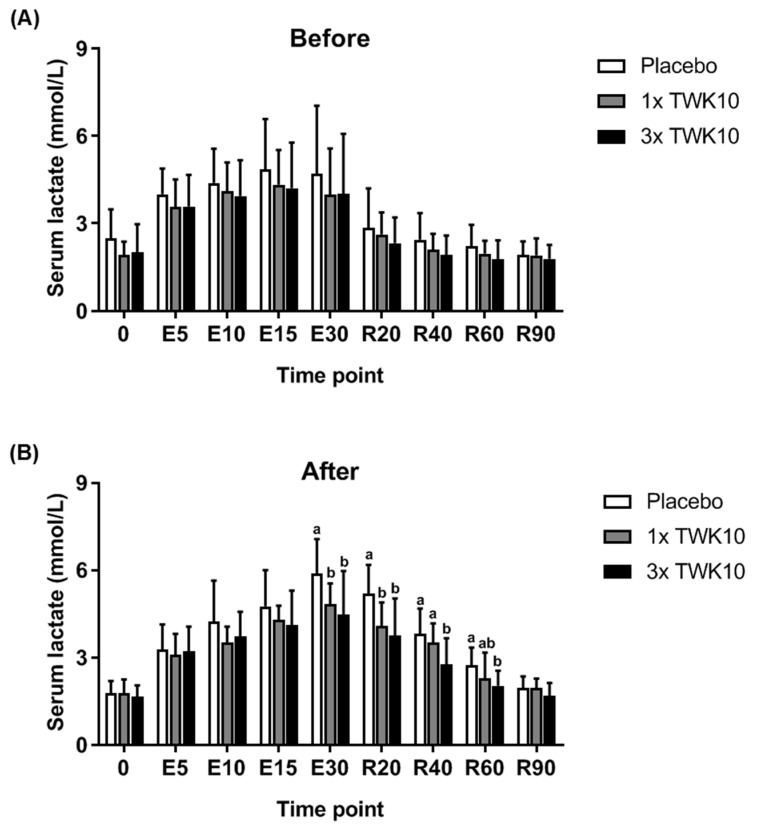
TWK10 reduced the content of serum lactate during and after exercise. E: Exercise; R: Rest. During fixed intensity and period exercise tests, blood samples were collected for lactate measurements at indicated time points. The contents of serum lactate were assessed (**A**) before and (**B**) after TWK10 administration. Data are shown as the mean ± SD. Statistical differences among groups were analyzed using the Tukey–Kramer test, and different letters (a, b) indicate a significant difference at *P* < 0.05.

As shown in Figure 3, the serum lactate increased as the exercise duration prolonged, reaching its highest level after 30 min of exercise intervention, and gradually decreased to the basal level through the recovery phase. Before administration, the alteration of circulating lactate during measurement periods was similar among groups (Figure 3A). However, in the TWK10 administration groups, lactate accumulation significantly improved either in the exercise phase (time point: E30) or in the recovery phase (time points: R20, R40, and R60) compared to the placebo group (Figure 3B). This beneficial effect exerted by TWK10 was stronger in the recovery phase.

**Figure 4:**
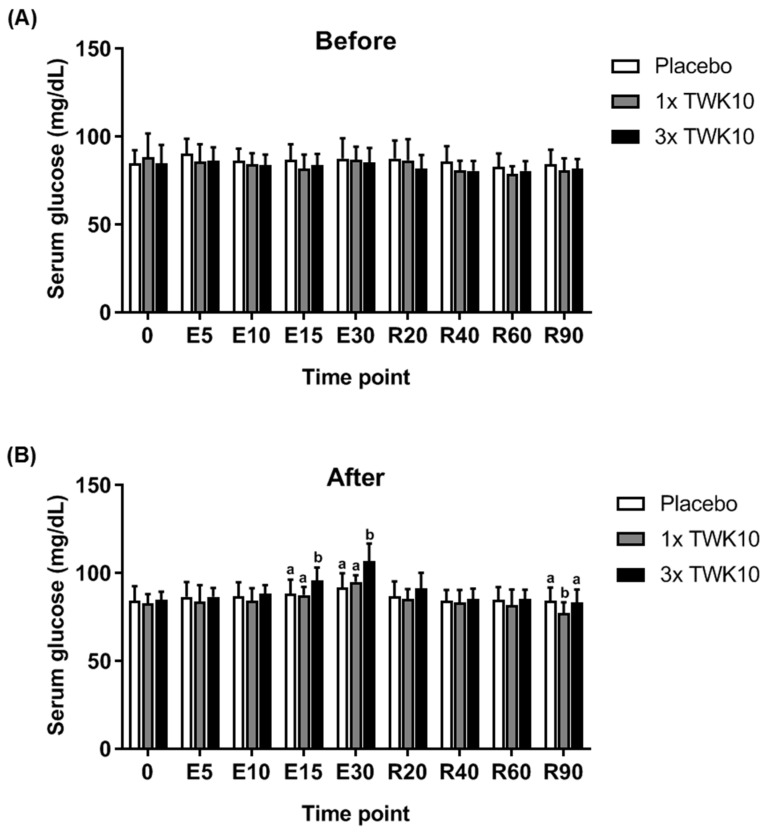
TWK10 reduced the content of serum glucose during and after exercise: (**A**) Before administration and (**B**) after administration. Data are shown as the mean ± SD, and the intergroup difference was analyzed by the Tukey–Kramer test. Different letters (a, b) indicate a significant difference at *P* < 0.05.

**Figure 5:**
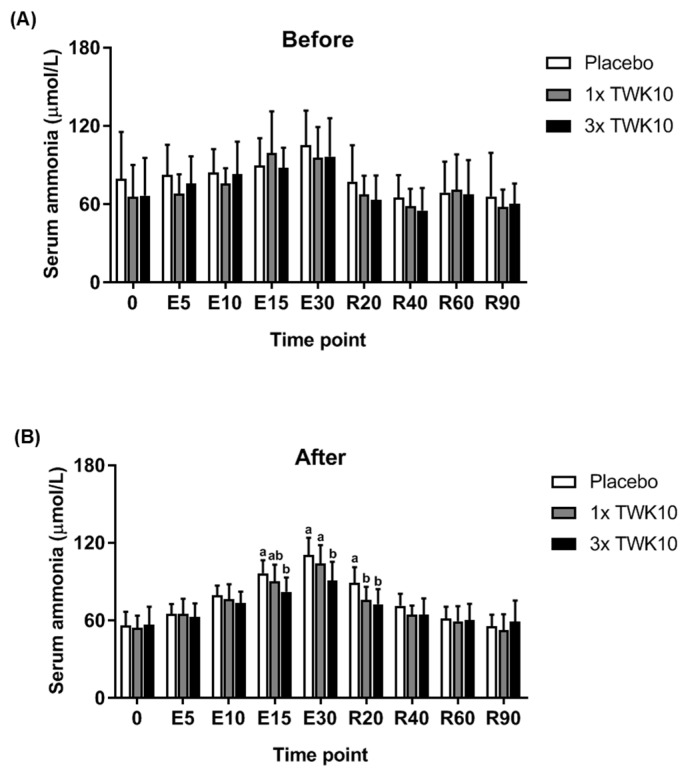
TWK10 reduced the content of serum ammonia during and after exercise. During fixed intensity and period exercise tests, blood samples were collected for ammonia measurements at indicated time points. The contents of serum ammonia were assessed (**A**) before and (**B**) after TWK10 administration. Data are shown as the mean ± SD. Statistical differences among groups were analyzed using the Tukey–Kramer test, and different letters (a, b) indicate a significant difference at *P* < 0.05.

For serum glucose levels, we observed no significant differences observed among groups before administration (Figure 4A). In the high-dose TWK10 group, the serum glucose level significantly increased during exercise stimulation (time points: E15 and E30) compared with the placebo group and was restored to basal levels in the recovery phase (Figure 4B). The alteration of serum ammonia (Figure 5) during exercise stimulation was similar to that of lactate (Figure 3). For the baseline of ammonia levels, no significant differences were observed among groups (Figure 5A). In TWK10-administered groups, especially the high-dose group (3× TWK10), the production of ammonia was significantly mitigated, not only during the exercise phase (time points: E15 and E30), but also during the recovery phase (time point R20) compared to those in the placebo group (Figure 5B). For the CK index, no significant difference was found among groups at the collection time points (data not shown).

### 3.3. Effects of TWK10 on Complete Blood Count Profiles

Blood samples were collected and analyzed at the 90 min rest point immediately after 30 min of exercise stimulation (60% VO_2max_). The CBC counts of the volunteers before and after TWK10 administration were obtained, and the ratio of the neutrophil count to lymphocyte count (NLR) and the ratio of the platelet count to lymphocyte count (PLR) were calculated according to indicated reference indices (Table 2). No significant differences in WBC, neutrophil, lymphocyte, monocyte, eosinophil, basophil, platelets, red blood cell (RBC), hemoglobin (Hb), hematocrit (Hct), mean corpuscular volume (MCV), mean corpuscular hemoglobin (MCH), and mean corpuscular hemoglobin concentration (MCHC) indices were detected among or within groups. Elevated NLR and PLR levels have been used as markers of exercise-induced systemic inflammatory responses [22]. In this study, no significant mitigation was observed in the NLR and PLR indices in the TWK10-administered groups compared with the placebo group.

### 3.4. Effects of TWK10 on Body Composition

**Figure 6:**
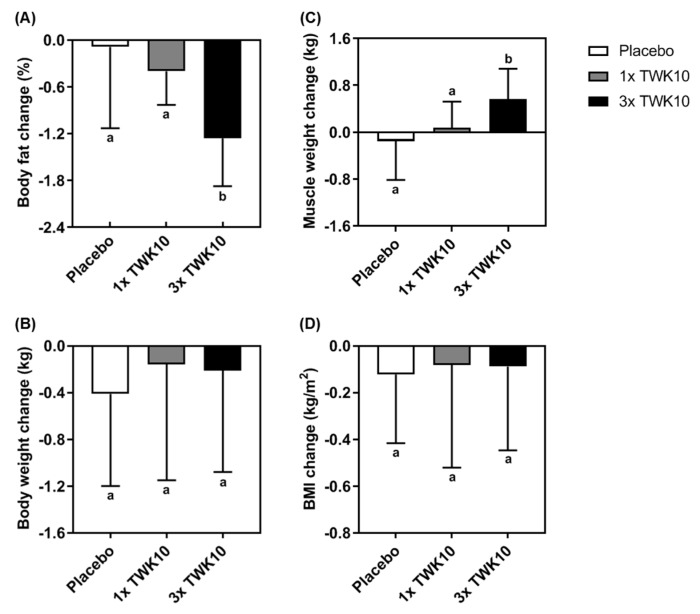
TWK10 exerted beneficial effects on body composition. The changes in (**A**) body fat, (**B**) body weight, (**C**) muscle weight, and (**D**) BMI were calculated as the difference between after and before administration and are shown as the mean ± SD. Statistical difference among groups was analyzed using the Kruskal–Wallis test, and different letters (a, b) indicate a significant difference at *P* < 0.05.

Body composition was assessed using the same bioelectrical impedance analyzer at the same time and under the same conditions to ensure consistency in the experimental processes. After six weeks of administration, the alteration in the body composition of each volunteer was monitored within groups. Body weight and BMI showed no significant differences within groups. Thus, we further analyzed the differences in changes before and after administration. TWK10-treated groups showed a fat mass decrease compared to the placebo treatment (Figure 6A). The results demonstrated that high doses of TWK10 (3× TWK10) significantly increased muscle mass change compared with the placebo group (Figure 6C).

### 3.5. Effects of TWK10 on Safety Assessment

The blood biochemical variables, including AST, ALT, BUN, CREA, UA, FFA, and glycerol, before (Figure S1) and after (Figure S2) six weeks of TWK10 administration were assessed at the baseline (time point 0), during exercise stimulation (time point E30), and in the recovery state (time point R90). No significant differences in these indices were found among groups.

### 3.6. Effects of TWK10 on Dietary Data

In terms of dietary information, we mainly recorded the caloric intake in both sexes before and after administration (Table 3). During TWK10 administration, the caloric intake did not show significant differences among groups in the two sexes.

## 4. Discussion

TWK10 was previously examined to elucidate its potential effects on energy homeostasis beneficial to exercise performance [18,21]. In this experiment, we enrolled a healthy population without regulatory training to investigate exercise performance, physiological adaptation, inflammation, body composition, and safety to further understand and validate the bio-efficacy of TWK10 for sport science application and health promotion.

Since 2017, probiotics have remained in the early stages of exercise-related research, especially for athletes, and only target immune function, airway improvement, and gastrointestinal function improvement [23]. Literature focused on athletic performance is lacking, and most published studies were on animal models. Exercise performance is an important functional index for evaluating the intervention effects of probiotics, and the methods generally applied to clinical trials are treadmill running and the cycle ergometer, which provide interchangeable references for monitoring training effects [24]. Smarkusz et al. also proposed that probiotics have potential applications for athletes, not only for disorders of the gastrointestinal tract and frequently recurrent upper respiratory tract infections, but also for physical capacity [25]. Four weeks of supplementation with a multi-strain probiotic increased athletes’ running time to fatigue in a hot environment [16]. In previous studies, *L. plantarum* PS128 was reported to maintain capacity after fatigue induction conditions [26]; in the current study, TWK10 was shown to lead to improvement in endurance capacity, possibly through different regulatory mechanisms. In the current study, we applied the treadmill method to evaluate the exercise performance before and after administration by individual VO_2max_ adjustment. Accordingly, six weeks of TWK10 administration significantly improved the exercise performance, which is consistent with our previous report where the exercise performance of the supplemental TWK10 group significantly increased by 36.76% compared with the placebo group [19] and the beneficial effect exerted by TWK10 occurred in a dose-dependent manner (Figure 2). While the 40% improvement in exercise performance appears reasonable and similar to previous human trials, more research is needed to confirm the effectiveness and mechanism of probiotic supplementation for improving athletic performance.

Lactate, an exercise-associated fatigue parameter, is produced from the glycolysis reaction [27]. The percentage of VO_2max_ exercise intensity plays a role in regulating the fuel mix of the proportions of carbohydrate (CHO) and fat oxidized by the working muscles. During low-intensity exercise (25% of VO_2max_), most of the energy requirements can be met from plasma fatty acid oxidation, and with a 65% exercise intensity of VO_2max_, fat contributes only about 50% of the total fuel requirements of exercise with the remainder of the energy obtained from CHO [28]. In the current study, we adjusted the individual appropriate exercise intensity (60% VO_2max_) to measure physiological adaptation. As shown in Figure 3B, TWK10 significantly decreased the production of serum lactate during exercise challenge and the recovery state. In a mouse model study, TWK-10 supplementation increased type I muscle fiber in gastrocnemius muscle [29]. Wilson et al. found that endurance training increases skeletal muscle mitochondria and type I fiber content and fatty acid oxidative capacity [30], which may explain the lower serum lactate due to the higher proportion of energy supplied through fatty acid oxidation instead of carbohydrates. Reduced serum lactate may be due to accelerating the clearance process as per Brook’s lactate shuttle theory [27]. During exercise, lactate is mainly produced in type II (fast twitch) muscle fibers, which use large amounts of glucose for energy, and it is cleared mainly by type I (slow twitch) muscle fibers. This is a complex process involving different lactate-specific transporters and enzymes; however, the process is efficient and exports less lactate to the blood (takes minutes) as it is cleared in higher amounts in the lactate-producing muscle, which takes seconds or milliseconds.

The microbial composition in athletes has been found to be distinct from normal people, defined by elevated abundances of *Veillonellaceae*, *Bacteroides*, *Prevotella*, *Methanobrevibacter*, or *Akkermansia* [31,32]. Scheiman et al. demonstrated that a strain of *Veillonella atypica* isolated from marathon runners could significantly elevate exhaustive treadmill run time via metabolic conversion of exercise-induced lactate to propionate [33]. Our previous animal study indicated that TWK10 could modulate the gut microbiota by altering the relative abundance of Firmicutes and Bacteroides (data not shown). Thus, the correlations of alterations of microbial composition and the exercise-improving effects exerted by TWK10 are worthy of further investigation.

The other index, ammonia, is also produced by the purine nucleotide cycle involved in muscle energy production, and excess ammonia can inhibit enzymes (pyruvate dehydrogenase and isocitrate dehydrogenase) in TCA oxidative metabolism. Fundamental differences in ammonia and adenylate metabolism exist between contracting type II (fast twitch) and type I (slow twitch) fibers [34]. Different skeletal muscle fiber types have different levels of adenylate deaminase activity, and muscles poor in mitochondria produce more ammonia during exercise than muscles rich in mitochondria [35,36]. This may explain our current finding that the TWK10-administered group showed lower serum ammonia due to the above-mentioned type I muscle increase in the mouse model [29].

Serum glucose is an important energy source for exercise that provides between 20% and 50% of the total oxidative energy production and between 25% and 100% of the total carbohydrate oxidized during submaximal exercise. The rate of serum glucose uptake decreases when serum-free fatty acid or muscle glycogen concentrations are very high [37,38,39]. In this study, serum glucose was elevated during the exercise phase (60% VO_2max_ exercise intensity) in TWK10-administered subjects (Figure 4B) and restored to levels comparable to the placebo control during the recovery phase. This may result from one or a combination of the following factors: Less glucose uptake by the muscle, less energy supplied through glucose, higher glycogenolysis, and higher gluconeogenesis during exercise. In our other mouse model study (unpublished), we found both liver and muscle glycogen increased in the TWK-10 administration group. This decreased the muscle uptake of serum glucose uptake. TWK-10 administration increased type I fibers, resulting in more energy supplied from fatty acid oxidation instead of glucose. Since we did not measure glycogenolysis in the liver and muscle in this experiment, we have no evidence of the amount of glucose formed from glycogenolysis. Regarding gluconeogenesis, we speculate that higher serum glucose from lactate produced during exercise was transported directly to the liver to be synthesized into glucose to flux the serum again, or through gut microbiota metabolic conversion to propionate and then synthesize glucose in the liver [33].

Low-level systemic inflammation can play important roles in initiating or exacerbating chronic diseases such as cardiovascular disease, obesity, osteoporosis, and geriatric cachexia. Therefore, the control of inflammation is crucial, and the effects of intervention have been validated by different strategies, including pharmacology, nutrition, and alternative medicine. Increases in appropriate physical activity potentially provide beneficial effects at a low cost, with ready availability, and fewer side effects for inflammation amelioration [40]. Regular exercise has been shown to mitigate inflammation induced by long-term cigarette smoking via regulation of immune, anabolic, and metabolic pathways [41]. Milajerdi et al. reviewed relevant studies and performed a meta-analysis of randomized clinical trials. They proposed that probiotic supplementation could significantly modulate inflammation via regulation of pro-inflammatory cytokines [42], which is consistent with our previous study [26]. In the current study, we applied the neutrophil to lymphocyte ratio (NLR) and platelet to lymphocyte ratio (PLR) as markers of systemic inflammation, as they have been widely used in previous studies [22]. We did not find any significant differences among the groups after TWK10 administration in the systemic inflammatory index after exercise intervention (Table 2). This may be due to applying only one short bout of moderate aerobic exercise, which did not induce any inflammation in the participants. 

Weight management has been a popular issue because the high prevalence of obesity is impacting public health [43]. Moderate and vigorous intensity exercise (50–70% of VO_2_ peak reserve) can significantly increase fat loss [44]. Aerobic exercise is an optimal method for reducing fat mass and body mass, but a program including resistance exercise is needed to increase lean mass in middle-aged overweight or obese individuals [45]. Thus, both fat loss and muscle gain are important goals for weight management and health promotion. In a nutrition and probiotics study, Bacillus probiotics were also reported to have protective effects against high-fat-diet-induced metabolic disorders in mice, and fat accumulation in subcutaneous and mesenteric tissues was significantly decreased by lipid oxidative gene activation and down-regulation of lipid uptake and lipogenesis [46]. Crovesy et al. systematically reviewed randomized controlled clinical trials and found that the effects of *Lactobacillus* on body weight and body fat in overweight subjects were strain-dependent [47]. We found that TWK10 supplementation had beneficial effects on the improvement of body composition in the current study (Figure 6), but not in the previous results on *L. plantarum* PS128 [26]. We think that TWK10 administration may increase weight management effects due to a synergistic effect on short chain fatty acid production and microbiota re-configuration.

In a study of obese postmenopausal women, the multispecies probiotic Ecologic^®^ Barrier (Winclove Probiotics, Amsterdam, Netherlands), including eight probiotics of *Lactobacillus* and *Bifidobacterium*, demonstrated a beneficial improvement in waist, fat mass, subcutaneous fat, uric acid, total cholesterol, triglycerides, low-density lipoprotein cholesterol, glucose, insulin, and the insulin-resistant index (HOMA-IR), as well as an increase in gut permeability to ameliorate sub-chronic low-grade systemic inflammation [48]. Thus, probiotics could be recognized as a complementary therapeutic agent for metabolic disorders. Probiotics could also exert anti-obesity activities possibly via gut microbiota alteration, energy metabolism remodeling, thermogenesis gene expression, glucose metabolism, and lipid metabolism to prevent obesity-associated disease [49]. In a systematic review and meta-analysis, probiotic supplementation was found to significantly reduce body weight, BMI, and fat percentage, but not fat mass [50]. In the current study, *L. plantarum* TWK10 was found to significantly benefit exercise performance and physiological adaption (Figure 2, Figure 3, Figure 4 and Figure 5), as well as body composition, especially in terms of fat and muscle change (Figure 6A,C) in a healthy young adult population. Thus, we believe *L. plantarum* TWK10 could be applied to an obese population for further validation of anti-obesity effects in future studies.

## 5. Conclusions

In the current study, we investigated the functional activities of TWK10 in a clinical trial to elucidate its benefits for sport science and health promotion. TWK10 significantly elevated the aerobic endurance capacity of non-trained healthy humans via the regulation of energy balance and metabolism for better physiological adaptation. We also observed improvements in body composition without specific exercise interventions. These probiotics, combined with exercise, could provide a practical strategy for health promotion and disease amelioration. Accordingly, the effects of TWK10, combined with different exercise types and intensities, could be further investigated in an overweight population to reveal its mechanisms in animal and clinical trials.

## Figures and Tables

**Table 1:** Background data of the subjects.

Characteristic	Placebo	1× TWK10	3× TWK10
**Male**	*n* = 9	*n* = 9	*n* = 9
Height (cm)	173.3 ± 3.6	175.6 ± 4.2	174.2 ± 7.3
Weight (kg)	70.6 ± 6.6	73.2 ± 10.1	77.6 ± 12.0
Age (y)	22.4 ± 1.8	22.8 ± 2.1	22.0 ± 1.7
BMI (m^2^/kg)	23.5 ± 2.2	23.8 ± 3.6	25.4 ± 2.8
VO_2max_ (mL/kg/min)	49.7 ± 7.1	49.8 ± 7.4	49.9 ± 8.3
**Female**	*n* = 9	*n* = 9	*n* = 9
Height (cm)	158.4 ± 6.2	160.2 ± 3.4	157.3 ± 5.1
Weight (kg)	50.3 ± 2.1	55.2 ± 5.2	49.7 ± 6.5
Age (y)	20.8 ± 1.0	21.2 ± 0.8	23.0 ± 5.2
BMI (m^2^/kg)	20.0 ± 1.2	21.5 ± 2.1	20.0 ± 2.0
VO_2max_ (mL/kg/min)	44.9 ± 8.6	44.8 ± 7.8	44.9 ± 9.5

Note: Values are represented as the mean ± SD, and intergroup differences were analyzed using the Tukey–Kramer test. There were no significant differences in the background data among the three groups.

**Table 2:** Blood count profiles of subjects.

Indicator	Placebo	1× TWK10		3× TWK10		
	Before	After	Before	After	Before	After
WBC (cumm)	7382 ± 1961	7357 ± 2350	8447 ± 2226	7924 ± 1898	7358 ± 1549	7063 ± 2043
Neutrophil (%)	69.0 ± 6.7	66.9 ± 9.6	70.9 ± 6.9	67.3 ± 8.2	63.2 ± 6.5	60.9 ± 8.4
Lymphocyte (%)	23.7 ± 6.0	25.6 ± 8.9	22.0 ± 5.4	24.7 ± 6.3	28.7 ± 6.6	29.7 ± 7.2
Monocyte (%)	5.35 ± 1.03	5.49 ± 1.25	5.17 ± 1.30	5.53 ± 1.64	5.80 ± 1.07	6.37 ± 2.51
Eosinophil (%)	1.42 ± 1.06	1.42 ± 0.96	1.40 ± 1.06	1.98 ± 1.77	1.67 ± 0.93	2.40 ± 1.30
Basophil (%)	0.58 ± 0.32	0.54 ± 0.25	0.47 ± 0.23	0.50 ± 0.23	0.63 ± 0.32	0.63 ± 0.31
RBC (MIL/cumm)	4.99 ± 0.54	5.02 ± 0.56	5.09 ± 0.55	5.07 ± 0.66	4.97 ± 0.61	5.04 ± 0.64
Hemoglobin (g/dL)	14.2 ± 1.9	14.2 ± 2.1	14.4 ± 1.3	14.4 ± 1.6	13.8 ± 1.4	13.8 ± 1.7
Hematocrit (%)	43.9 ± 5.1	44.1 ± 4.9	44.6 ± 3.5	44.4 ± 4.1	42.7 ± 3.4	43.4 ± 4.3
MCV (fl)	88.3 ± 8.0	88.1 ± 8.7	88.0 ± 6.6	88.1 ± 7.1	86.7 ± 8.9	86.8 ± 7.9
MCH (pg)	28.5 ± 3.2	28.3 ± 3.4	28.4 ± 2.5	28.5 ± 2.5	28.0 ± 3.3	27.6 ± 3.3
MCHC (%)	32.2 ± 1.3	32.0 ± 1.4	32.2 ± 0.9	32.3 ± 1.1	32.2 ± 1.1	31.8 ± 1.3
Platelet (10^3^/cumm)	258 ± 61	258 ± 70	280 ± 57	274 ± 58	243 ± 62	238 ± 66
NLR	3.14 ± 0.98 ^a^	3.12 ± 1.62	3.50 ± 1.28 ^a^	2.99 ± 1.11	2.34 ± 0.68 ^b^	2.25 ± 0.89
PLR	165 ± 62 ^a,b^	149 ± 60	159 ± 27 ^a^	150 ± 34	121 ± 34 ^b^	122 ± 36

Note: Data are shown as the mean ± SD. Intergroup differences before and after administration were analyzed using the Tukey–Kramer test. Statistical differences in the ratio of the neutrophil count to the lymphocyte count (NLR) and ratio of platelets to lymphocytes (PLR) were analyzed using the Kruskal–Wallis test. Values for before administration with different superscripts (a, b) within the same row indicate a significant difference at *P* < 0.05. No significant differences in the data were found after administration amongst the three groups.

**Table 3:** Dietary information of subjects.

	Before Administration			After Administration		
	Placebo	1× TWK10	3× TWK10	Placebo	1× TWK10	3× TWK10
**Men**	*n* = 9	*n* = 9	*n* = 9	*n* = 9	*n* = 9	*n* = 9
Carbohydrates (g/day)	298 ± 29	290 ± 40	292 ± 54	281 ± 45	288 ± 36	285 ± 43
Protein (g/day)	110.3 ± 16.2	98.9 ± 11.2	102.9 ± 20.0	95.1 ± 17.1	92.5 ± 19.3	90.9 ± 22.9
Lipid (g/day)	117.1 ± 16.0	110.6 ± 13.8	111.8 ± 16.5	105.7 ± 9.3	104.5 ± 28.2	99.8 ± 15.9
Calories (kcal/day)	2686 ± 199	2268 ± 865	2585 ± 388	2456 ± 285	2464 ± 414	2401 ± 278
**Women**	*n* = 9	*n* = 9	*n* = 9	*n* = 9	*n* = 9	*n* = 9
Carbohydrates (g/day)	204 ± 31	200 ± 29	195 ± 19	200 ± 17	206 ± 27	206 ± 15
Protein (g/day)	68.1 ± 13.6	69.5 ± 7.7	66.1 ± 8.2	67.1 ± 14.8	68.6 ± 16.8	65.0 ± 10.2
Lipid (g/day)	82.6 ± 14.2	83.2 ± 12.4	80.8 ± 5.9	84.5 ± 14.6	85.6 ± 12.6	81.2 ± 12.2
Calories (kcal/day)	1831 ± 254	1826 ± 165	1770 ± 123	1827 ± 166	1867 ± 138	1814 ± 145

Note: Values are shown as the mean ± SD: Before administration and after administration, and intergroup difference was analyzed using the Tukey–Kramer test. There were no significant differences in the dietary information amongst the three groups.

## Supplementary Materials

The following are available online at https://www.mdpi.com/2072-6643/11/11/2836/s1, Figure S1: Blood biochemical profiles in the different time points of the subjects before the administration of TWK10: (A) AST; (B) ALT; (C) BUN; (D) Creatinine; (E) UA; (F) Free fatty acids; (G) Glycerol. 0, time zero; E30, exercise for 30 min; R90, resting 90 min after exercise. Statistical difference among groups was analyzed by Tukey–Kramer test at the same time point. Figure S2: Blood biochemical profiles in the different time points of the subjects after the administration of TWK10: (A) AST; (B) ALT; (C) BUN; (D) Creatinine; (E) UA; (F) Free fatty acids; (G) Glycerol. 0, time zero; E30, exercise for 30 min; R90, resting 90 min after exercise. Statistical difference among groups was analyzed by Tukey–Kramer test at the same time point.
