# Supplementary material for: Effect of Lactobacillus plantarum TWK10 on Exercise Physiological Adaptation, Performance, and Body Composition in Healthy Humans
Source: Nutrients. 2019 Nov 19;11(11):2836. doi: 10.3390/nu11112836 (PMC6893516; doi:10.3390/nu11112836)
Supplement: Supplementary file 1 [file nutrients-11-02836-s001.pdf]

Supplementary figures

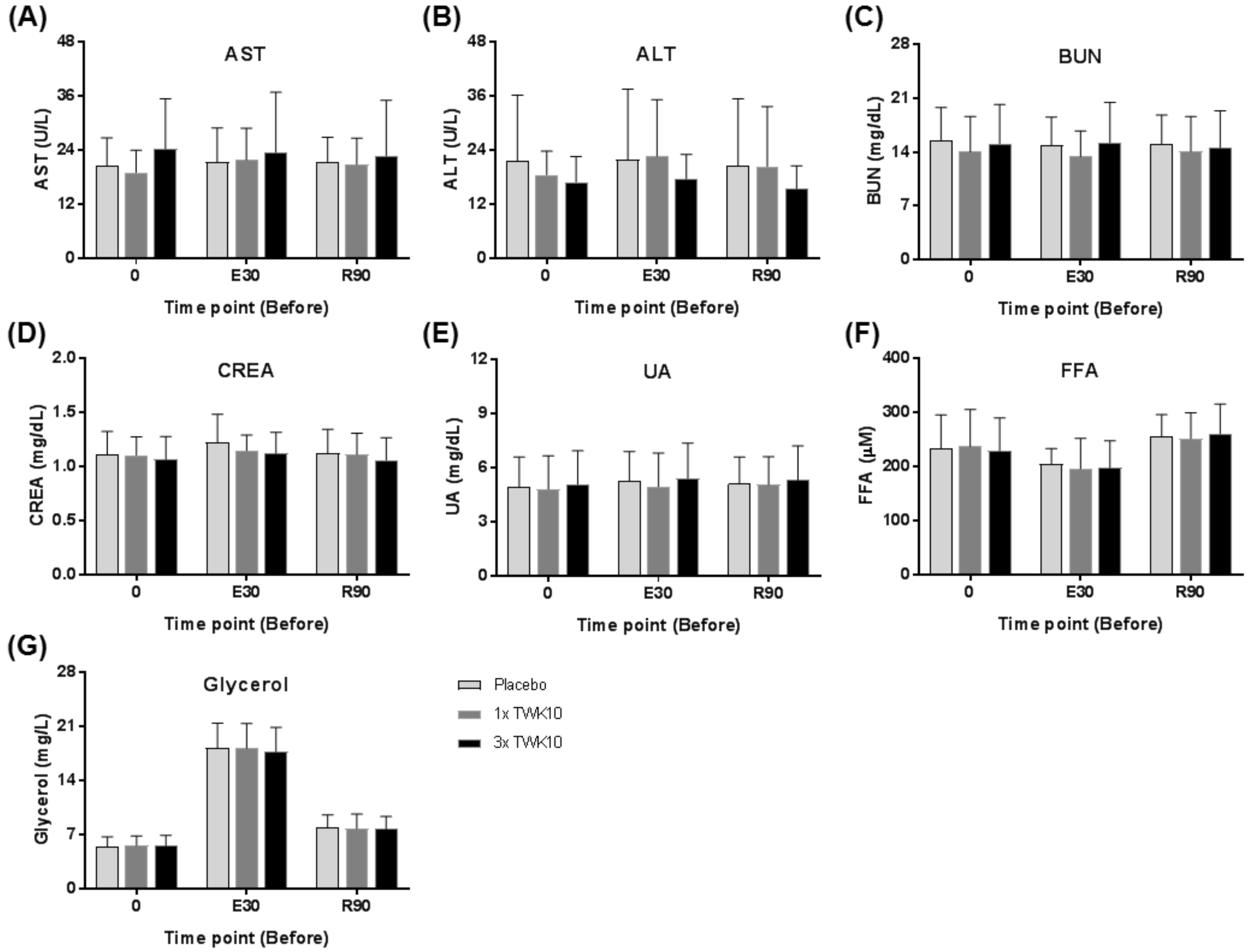

Figure S1. Blood biochemical profiles in the different time points of the subjects before the administration of TWK10: **(A)** AST; **(B)** ALT; **(C)** BUN; **(D)** Creatinine; **(E)** UA; **(F)** Free fatty acids; **(G)** Glycerol. 0, time zero; E30, exercise for 30 min; R90, resting 90 min after exercise. Statistical difference among groups was analyzed by Tukey–Kramer test at the same time point.

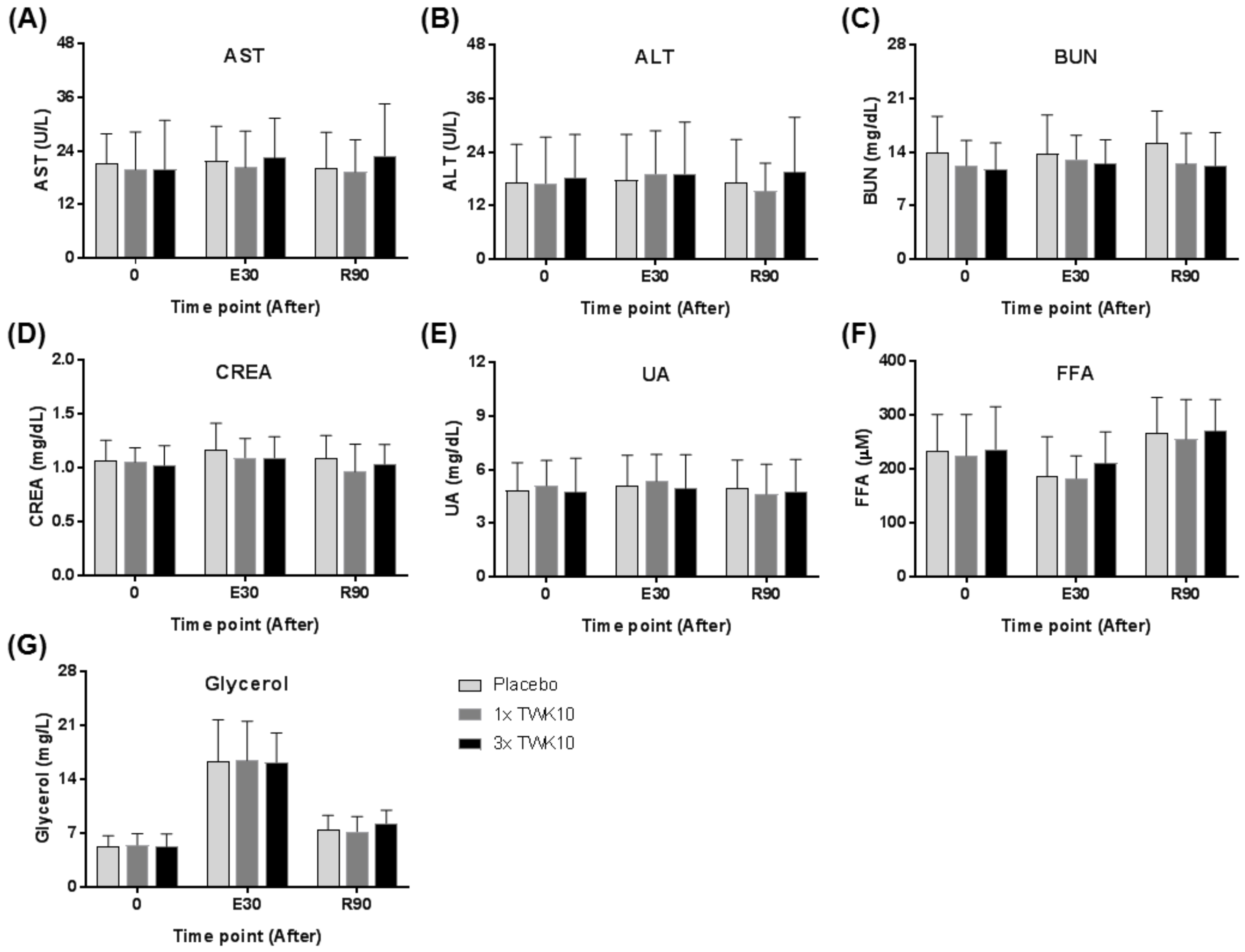

Figure S2. Blood biochemical profiles in the different time points of the subjects after the administration of TWK10: **(A)** AST; **(B)** ALT; **(C)** BUN; **(D)** Creatinine; **(E)** UA; **(F)** Free fatty acids; **(G)** Glycerol. 0, time zero; E30, exercise for 30 min; R90, resting 90 min after exercise. Statistical difference among groups was analyzed by Tukey–Kramer test at the same time point.
